# Supplementary figures and images for: FENCE: Flexible Electric Noise Reduction Endo‐Shield for the Suppression of Electromagnetic Interference in Low‐Field MRI
Source: NMR Biomed. 2026 Apr 26;39(6):e70287. doi: 10.1002/nbm.70287 (PMC13111743; doi:10.1002/nbm.70287)

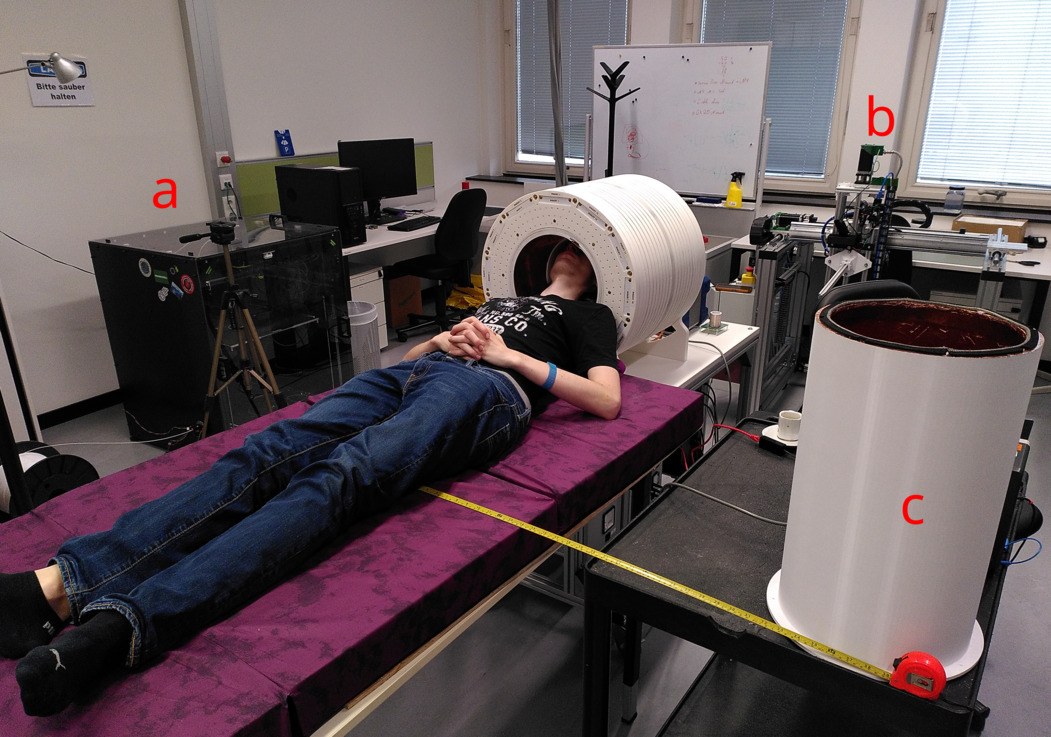

Supplement: Supplementary file 1 — Figure S1: Measurement setup for the in vivo measurements. Different EMI sources are highlighted in red. (a) shows the 3D printer (b) the field mapping robot and (c) the copper foil cylinder for EMI coupling. Figure_S1.jpg [file NBM-39-e70287-s001.jpg]
